# Nuclear factor 90 uses an ADAR2-like binding mode to recognize specific bases in dsRNA

**DOI:** 10.1093/nar/gkv1508

**Published:** 2015-12-27

**Authors:** Uma Jayachandran, Heather Grey, Atlanta G. Cook

**Affiliations:** 1 Wellcome Trust Centre for Cell Biology, University of Edinburgh, Michael Swann Building, Max Born Crescent, Edinburgh EH9 3BF, UK; 2 Institute of Molecular Plant Sciences, University of Edinburgh, Daniel Rutherford Building, Max Born Crescent, Edinburgh EH9 3BF, UK

## Abstract

Nuclear factors 90 and 45 (NF90 and NF45) form a protein complex involved in the post-transcriptional control of many genes in vertebrates. NF90 is a member of the dsRNA binding domain (dsRBD) family of proteins. RNA binding partners identified so far include elements in 3′ untranslated regions of specific mRNAs and several non-coding RNAs. In NF90, a tandem pair of dsRBDs separated by a natively unstructured segment confers dsRNA binding activity. We determined a crystal structure of the tandem dsRBDs of NF90 in complex with a synthetic dsRNA. This complex shows surprising similarity to the tandem dsRBDs from an adenosine-to-inosine editing enzyme, ADAR2 in complex with a substrate RNA. Residues involved in unusual base-specific recognition in the minor groove of dsRNA are conserved between NF90 and ADAR2. These data suggest that, like ADAR2, underlying sequences in dsRNA may influence how NF90 recognizes its target RNAs.

## INTRODUCTION

Nuclear factor 90 (NF90) is a double-stranded RNA (dsRNA) binding protein, conserved in vertebrates, which affects gene expression at transcriptional, post-transcriptional and translational levels (1–3). Also known as interleukin enhancer binding factor 3 (ILF3), NF90 is reported to affect post-transcriptional stability and/or translation of specific mRNAs, to alter miRNA processing and to interact with the nuclear export machinery (4–8). Several (+)-stranded RNA viruses such as hepatitis C virus and Dengue virus use NF90 as a host factor (9–11). However, at present, there is no clear mechanistic understanding of how NF90 performs these various roles at a molecular level.

**Figure 1.**
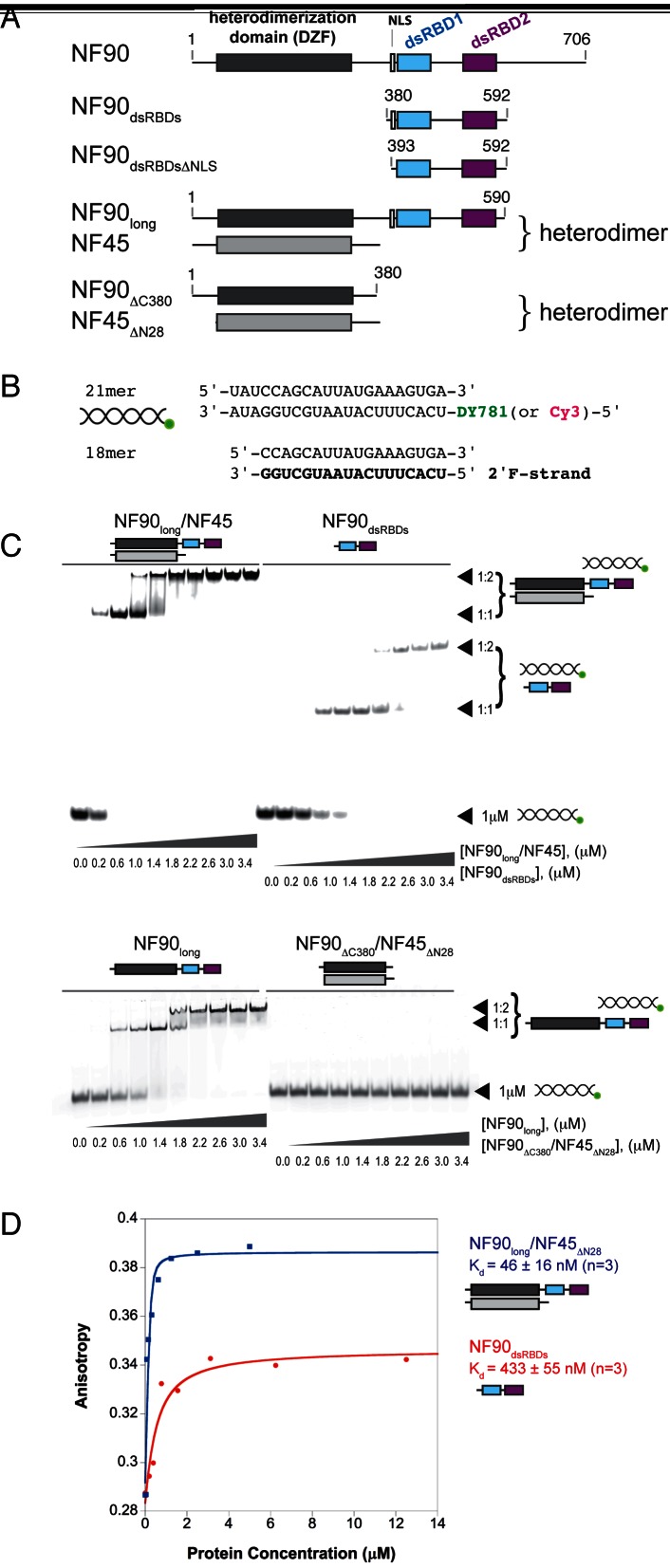
NF90 constructs bind to dsRNA with a 1:2 protein:RNA ratio. (**A**) Schematic of mouse NF90 domain structure, indicating the constructs used in this study. (**B**) Overview of 21mer and 18mer dsRNA constructs used in RNA binding assays and co-crystallization with NF90_dsRBDs_. The 18mer strand that contains 2′ fluorine is shown in bold. (**C**) Electrophoretic mobility shift assay of DY781-labelled 21mer RNA titrated with NF90 constructs and NF90/NF45 complexes. Protein concentration is shown below the gel. (**D**) Fluorescence anisotropy of Cy3-labelled 21mer dsRNA was used to measure dissociation constants of NF90_long_/NF45_ΔN28_ and NF90_dsRBDs_. Solid circles and squares show anisotropy values for samples titrated with NF90_dsRBDs_ and NF90_long_/NF45_ΔN28_, respectively. Solid lines indicate best fit to the data from which *K*_d_ values were calculated. *K*_d_ values are given as the mean of three independent experiments ± standard deviation. Raw data from a representative experiment are shown with the calculated fitted curves.

NF90 (and its brain- and testes-specific paralogue, spermatid perinuclear RNA binding protein, SPNR (12)) consists of three structured domains followed by a C-terminal region that is predicted to be natively unstructured (Figure 1A). The first domain, annotated as a ‘domain associated with zinc fingers’ or DZF domain, has a nucleotidyltransferase fold and mediates heterodimerization with a structurally similar domain in nuclear factor 45 (NF45) (13). Downstream of the DZF domain there is a nuclear localization signal (NLS) followed by a tandem pair of double stranded RNA binding domains (dsRBDs) separated by a 52 amino acid linker sequence that is predicted to be natively unstructured (Figure 1A).

DsRBDs (also known as dsRNA binding motifs or dsRBMs) are widespread in proteins involved in many aspects of RNA metabolism (14). They are 65–70 amino acids long and generally recognize dsRNA through shape complementarity and electrostatic interactions with the RNA backbone, rather than sequence-specific interactions with RNA bases (14,15). Some dsRBD-containing proteins such as the adenosine-to-inosine (A-to-I) deaminases acting on RNA (ADARs) are known to bind specific RNAs in cells. ADARs are RNA modifying enzymes that catalyse the hydrolytic deamination of adenosine to inosine (16,17). Inosine has a different base pairing pattern to adenine and so is read as guanine by the translation and splicing machineries. A-to-I editing occurs in the nucleus on pre-mRNAs and non-coding RNAs. It can change the encoded proteins sequence (recoding), alter splice sites and change seed regions in miRNAs (16,17). A well-characterized interaction between an ADAR protein and its RNA substrate is mammalian ADAR2 with *gluA2* pre-mRNA (18,19). GluA2 is an ion channel that is recoded at two codons, known as the Q/R and R/G sites (20). The ADAR2 dsRBDs direct the catalytic domain by docking on RNA hairpin structures that form between exonic and intronic sequences (21). Solution studies of ADAR2 dsRBDs with the GluA2(R/G) hairpin fragment revealed that ADAR2 makes base-specific interactions in the minor groove, showing that some dsRBDs are more sequence selective than previously thought (22).

To better understand the RNA recognition properties of NF90, we solved the crystal structure of the tandem dsRBDs of NF90 with a synthetic dsRNA. Surprisingly, NF90 tandem dsRBDs have high structural similarity to ADAR2 dsRBDs and show similar base-specific interactions with a G-X_n_-A motif in the minor groove. We further show that dsRNA fragments lacking the preferred G-X_n_-A motif are poor competitors of dsRNA binding. The dsRBD domains of NF90 only contribute part of the RNA binding activity of this protein, with a higher affinity for dsRNA found in the full-length NF90/NF45 complex. The similarity to ADAR2 suggests that NF90 is likely to recognize partner RNAs in a highly specific manner, consistent with observations that NF90 has important roles in post-transcriptional regulation of gene expression.

## MATERIALS AND METHODS

### Protein expression and purification

Two constructs of mouse NF90 were used for electrophoretic mobility shift assays (EMSA) (NF90_dsRBDs_, residues 380–590) and for crystallization and isothermal titration calorimetry (ITC) (NF90_dsRBDsΔNLS_, residues 393–592) (Figure 1A). These constructs were expressed as glutathione-S transferase (GST)-fusion proteins in *Escherichia coli* strain BL21(*DE3*) and induced overnight with 1 mM isopropyl-β-D-1 thiogalactopyranoside (IPTG) at 18°C. Cells were lysed using a cell disruptor (Constant Systems) in 40 mM HEPES pH 7.5, 150 mM NaCl, 1 mM dithiothreitol (DTT), supplemented with a protease inhibitor cocktail (Roche) and DNAse I (Sigma Aldrich). Cleared lysates were bound in batch to GSH resin (GE Healthcare) at 4°C, packed into a chromatography column and eluted in 40 mM HEPES pH7.5, 150 mM NaCl, 1 mM DTT and 20 mM reduced glutathione. The protein was dialysed into 20 mM HEPES pH7.5, 50 mM NaCl overnight with 1 mg Tobacco etch virus (TEV) protease. TEV protease and GST were separated from NF90 using heparin sepharose or mono S cation exchange chromatography and eluted with a salt gradient to 1M NaCl. The protein was further purified by size exclusion chromatography using a Superdex S75 column (GE Healthcare). NF90_long_ (residues 1–590 of mouse NF90) and NF45 (full length or residues 28–390) were expressed separately in BL21(*DE3*) cells as N-terminally 6×His tagged proteins, induced at 18°C overnight and co-lysed in 20 mM Tris.HCl pH 8.0, 500 mM NaCl, 10 mM imidazole (pH 8.0) and 1 mM β-mercaptoethanol with protease inhibitor cocktail. Tagged proteins were extracted from clarified lysates by batch binding to Ni^2+^-NTA resin, packed into a column and eluted with a gradient from 0.01–1M imidazole, pH 8.0. Eluted proteins were dialysed overnight into 20 mM Tris.HCl pH 7.5, 50 mM NaCl, 1 mM DTT with TEV protease to remove the tags. NF90_long_ and NF45 were mixed and the complex was purified by heparin sepharose chromatography and size exclusion chromatography in 20 mM Tris.HCl pH 7.5, 150 mM potassium acetate, 1 mM DTT. NF90_long_ alone was purified using the same conditions as the NF90_long_/NF45 complex. NF90_ΔC380_/NF45_ΔN28_ were purified as described previously (13).

### RNA preparation

18mer 2′F modified RNA was a kind gift from ISIS pharmaceuticals (Carlsbad, USA)(23). Duplexes were made by mixing 2′F modified or unmodified RNA (UCACUUUCAUAAUGCUGG) with an equal concentration of a complementary sequence, heating to 95°C for 5 min and cooling to room temperature overnight. For EMSA assays, a 21mer RNA labelled with a 5′ fluorescent DY781 dye and containing a three base extension to the 18mer sequence was used (5′-DY781-UCACUUUCAUAAUGCUGGAUA, Biomers GmbH). Oligonucleotides were reconstituted in H_2_O, mixed at a 1:1.2 molar ratio of labelled to unlabelled strand for a final concentration of 100 μM duplex and annealed overnight as for 18mer duplexes. For competition assays, GluA2(R/G) RNA was synthesized by Biomers GmbH and reconstituted in water to a final concentration of 100 μM (CAUUAAGGUGGGUGGAAUAGUAUAACAAUAUGCUAAAUGUUGUUAUAGUAUCCCACCUACCCUGAUG). GluA2(R/G) RNA was annealed by heating to 95°C for 3 min and snap cooled on ice for 30 min. Duplexes for unlabelled 17mer RNA (UCACUUUCAUAAUGCUG) and GC- and AU-rich duplexes were prepared as for 18mer duplexes.

### Crystallization and structure solution

A protein–RNA complex was formed by mixing a 1:1.2 ratio of protein to RNA followed by size exclusion chromatography to remove excess RNA. The complex was concentrated to an optical density at 280 nm of 15 absorbance units. Initial sparse matrix screening was carried out with NF90_dsRBDs_–18mer RNA complexes. Crystals were grown in sitting drops in 50 mM sodium citrate pH 5.0, 1.0M ammonium formate. Crystals grown from NF90_dsRBDsΔNLS_–18mer RNA complexes showed improved diffraction quality. Crystals were cryoprotected in mother liquor supplemented with 30% glycerol and flash cooled to 100 K. Data were collected at beam line I03 at Diamond Light Source using a PILATUS detector. Data were indexed using XDS and scaled and merged using SCALA (24,25). Phases were obtained using a calculated model of A-form RNA from COOT (26) and PDB ID: 3P1X (27) as search models in molecular replacement with PHASER (28). Due to the lower resolution of the data and the structural similarity between native and 2′F modified RNA, both RNA chains were modelled as native RNA (29). DsRBD1 from SPNR (PDB ID: 2DMY (30)), was manually fitted into the remaining electron density. The structure was refined using PHENIX (31) and rebuilt using COOT (26).

### Electrophoretic mobility shift assays

Proteins were dialysed into 20 mM HEPES pH 7.5 and 150 mM potassium acetate. Binding reactions, containing 1 μM labelled RNA and increasing concentrations of NF90_380_–_592_ or NF90_long_/NF45 in a 10 μl reaction volume, were incubated on ice for 30–45 min. A 6% polyacrylamide gel in 0.5× Tris/Borate/EDTA buffer (TBE) was pre-run at 2W at 4°C for 1 h. Samples were mixed with 2 μl of native gel loading buffer and 4 μl was loaded onto the gel. After 2 h at 2W, the gel was scanned on a LICOR Odyssey fluorescent infrared scanner at 800 nm. Images were converted to greyscale and inverted using ImageJ. Competition assays were carried out in a 10 μl reaction volume. A 1:1 molar ratio complex of 21mer dsRNA with NF90_long_/NF45 was made by pre-incubation on ice for 1 h. The complex was used at a final concentration of 0.5 μM with buffer and/or increasing concentrations of unlabelled competitor RNA.

### Isothermal titration calorimetry (ITC)

NF90_dsRBDsΔNLS_ and 18mer RNA (either native or 2′F modified) were dialysed overnight in 20 mM HEPES pH 7.5 and 50 mM NaCl. Protein and RNA concentrations were determined using 280 nm absorbance on a Nanodrop spectrophotometer. Measurements were made at 25°C using a Microcal Auto-iTC200 system (GE Healthcare). A total of 300 μM NF90_dsRBDs_ in the syringe was titrated into 12 μM 18mer dsRNA in the cell with a total of 16 × 2.5 μl injections and 3 min between each injection. To control for dilution, NF90_dsRBΔsΔNLS_ was titrated into buffer alone. Experiments were repeated three times and *K*_d app_ values were obtained using the Microcal iTC200 software.

### Fluorescence anisotropy

A total of 250 nM Cy3-labelled 21mer dsRNA (in 20 mM HEPES pH 7.5, 150 mM potassium acetate) was incubated with increasing concentrations of mNF90_dsRBDs_ (residues 380–592) or mNF90_long_(residues 1–592)-mNF45_ΔN28_ for 20 min at 23.5°C. Anisotropy was measured on a Spectramax M5 multi-plate reader (Molecular Devices) using 530 and 562 nm wavelengths for excitation and emission, respectively. Experimentally observed anisotropies as a function of protein concentration were fit to equation 6 (from (32)) to determine the equilibrium dissociation constant, *K*_d_, for binding of the labelled RNA duplex to mNF90 constructs (KaleidaGraph V4.03, Synergy Software). Binding constants were determined from three independent experiments, from which mean *K*_d_ and standard deviations were calculated.

## RESULTS

### NF90/NF45 complexes form monomers and dimers on dsRNA

While NF90 has been shown to bind to a number of mRNAs and ncRNAs (33–37), there is limited information about what constitutes a minimal RNA binding motif for NF90. We made use of a previous study by Rigo *et al*. (23). They showed that NF90/NF45 complexes are recruited to mRNA transcripts in cells transfected with complementary 18mer oligonucleotides uniformly modified at the 2′ ribose position with fluorine (2′F) (23). We expressed and purified different NF90 constructs (Figure 1A, Supplementary Figure S1) and characterized their RNA binding activity with a 21mer dsRNA containing the same sequence as used in the study by Rigo *et al*., but extended at the 5′ end by 3 bases (Figure 1B). In electrophoretic mobility shift assays with labelled 21mer dsRNA, both constructs containing the dsRBD domains (NF90_dsRBDs_, residues 380–592) and longer constructs containing all folded domains of NF90 (NF90_long_, residues 1–590) and NF90_long_/NF45 complex were able to shift the labelled dsRNA (Figure 1C). However, the heterodimerization domain (NF90_ΔC380_/NF45_ΔN28_) is unable to interact with dsRNA. NF90_long_/NF45, NF90_long_ and NF90_dsRBDs_ show the formation of two distinct RNA–protein complexes at 1:1 and 1:2 RNA:protein (Figure 1C). Although these complexes are qualitatively similar, NF90_long_/NF45_ΔN28_ and NF90_dsRBDs_ differ significantly in their affinity for dsRNA: fluorescence anisotropy assays using Cy3-labelled 21mer dsRNA show that NF90_dsRBDs_ has a *K*_d_ of 433 ± 55 nM while the longer form of the complex binds with a *K*_d_ of 46 ± 16 nM (Figure 1D). This ∼10-fold difference in affinity indicates that other parts of NF90 contribute to dsRNA binding.

To further characterize interactions between NF90 and the shorter 18mer dsRNA, we co-crystallized NF90_dsRBDs_ with 18mer duplex RNA with 2′F ribose on one strand. Complex formation was observed using size exclusion chromatography, where the protein–RNA complex eluted earlier than the protein or RNA alone (Supplementary Figure S1B). Crystals of the NF90–RNA complex showed improved diffraction properties when a shorter construct of NF90_dsRBDs_ lacking the NLS was used (NF90_dsRBDsΔNLS_, Figure 1A). The crystals diffracted to 3.5 Å in space group *P*3_1_2. No crystals were obtained with complexes with unmodified RNA. ITC measurements indicate that NF90_dsRBDsΔNLS_ minimal construct binds to both modified and unmodified RNA with similar binding constants (*K*_d,app_) of ∼2.4 mM (Supplementary Figure S1C). This indicates that a difference in affinity is not the reason for the difference in their ability to crystallize. Previous observations show that duplexes containing 2′F modified RNAs have higher thermal stability than native RNA molecules and are more resistant to degradation, which may explain the difference between native and modified dsRNA in crystallization experiments (29,38). Consistent with our EMSA assays, ITC assays consistently show a binding ratio of 1:2 RNA to protein.

The crystal structure was solved by molecular replacement using a calculated model of A-form RNA, with 2′F modified RNA modelled as native RNA, and a crystal structure of human NF90 dsRBD2 as search models (PDB ID: 3P1X (27)). DsRBD1 was fitted manually using the coordinates of dsRBD1 from SPNR, which has 100% sequence identity in this domain (PDB ID: 2DMY (30)). After model building and refinement the final model shows good stereochemistry with R_work_ = 23.9% and R_free_ = 27.7% (Table 1).

**Table 1.** X-ray crystallographic data collection and refinement statistics

**Data collection**	
Beamline	Diamond light source I03
Wavelength (Å)	0.979
Space group	*P*3_1_ 1 2
Unit cell (Å)	a = b = 100.1, c = 107.8 α = β = 90° γ = 120°
Resolution (High resolution shell)	50–3.5 (3.69–3.5)
Unique reflections	7981
Completeness (%)	99.9 (100)
Multiplicity	14.9
I/σ(I)	22.1 (3.6)
R_merge_	0.09 (0.91)
**Refinement**	
R_free_	27.3%
R_work_	23.9%
No. of atoms	1637
No. of protein atoms	879
No. of nucleic acid atoms	757
r.m.s bond angles	1.12°
r.m.s bond distances	0.010 Å
Average B factors	
nucleic acid	136.7 Å^2^
protein	126.5 Å^2^
Ramachandran values	
Favoured	90%
Additionally allowed	9%
Unfavourable	1%

### NF90 dsRBDs bind to a continuous RNA double helix in the crystal

**Figure 2.**
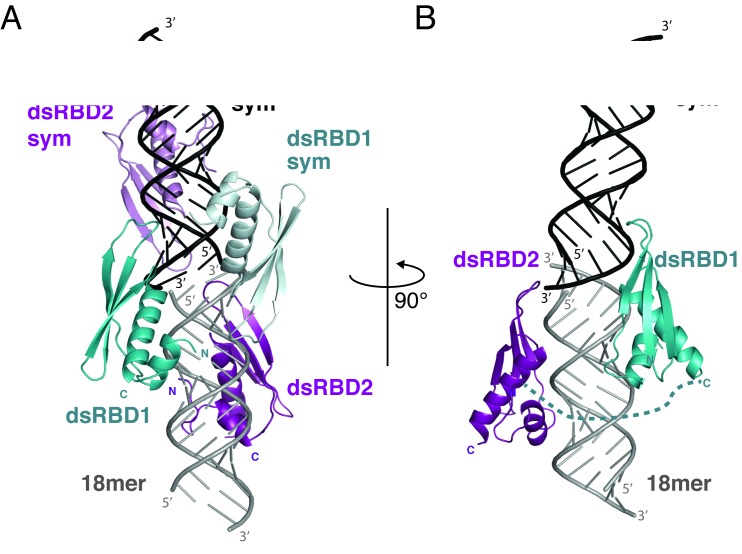
Structural overview of NF90_dsRBDsΔNLS_ in complex with dsRNA. (**A**) A view of the NF90_dsRBDsΔNLS_–18mer RNA complex and a symmetry equivalent complex shown as cartoon. The asymmetric unit consists of 18mer RNA (light grey), dsRBD1 (blue) and dsRBD2 (purple). Symmetry equivalent domains are shown in light blue and violet with associated RNA in black. (**B**) An orthogonal view of the complex with symmetry equivalent protein domains removed for clarity. The linker between the domains is represented as a dotted line.

In the structure of NF90_dsRBDsΔNLS_ in complex with 18mer RNA the two dsRBDs bind either side of the dsRNA (Figure 2A and B). All bases of the RNA are observed in the electron density map. The RNA helix extends through the crystal lattice by co-axial stacking with neighbouring duplexes (Supplementary Figure S2A). An RNA–RNA interface generates the 2-fold crystallographic axis, across which symmetry equivalents of dsRBD1 are bound (Figure 2A). A slight deviation of the central axes of the dsRNA occurs between the two duplexes at this binding site, suggesting that dsRBD1 has a preference for flexible or intrinsically curved RNA structures.

**Figure 3.**
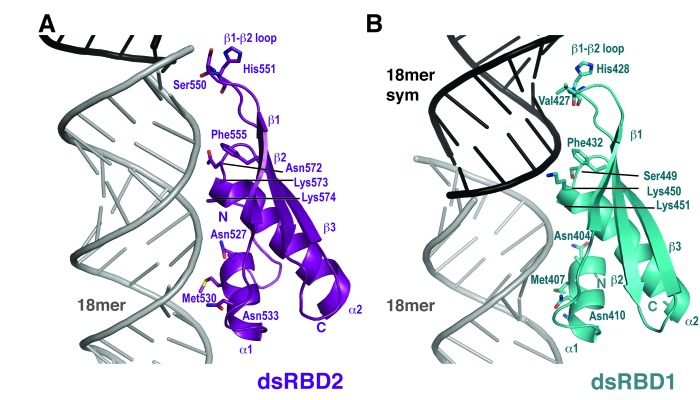
Interactions of NF90 side chains with dsRNA. Close-up view of (**A**) dsRBD2 (purple) and (**B**) dsRBD1 (blue) showing interactions with RNA. Side chains of residues that interact directly with the RNA are shown as sticks.

**Figure 4.**
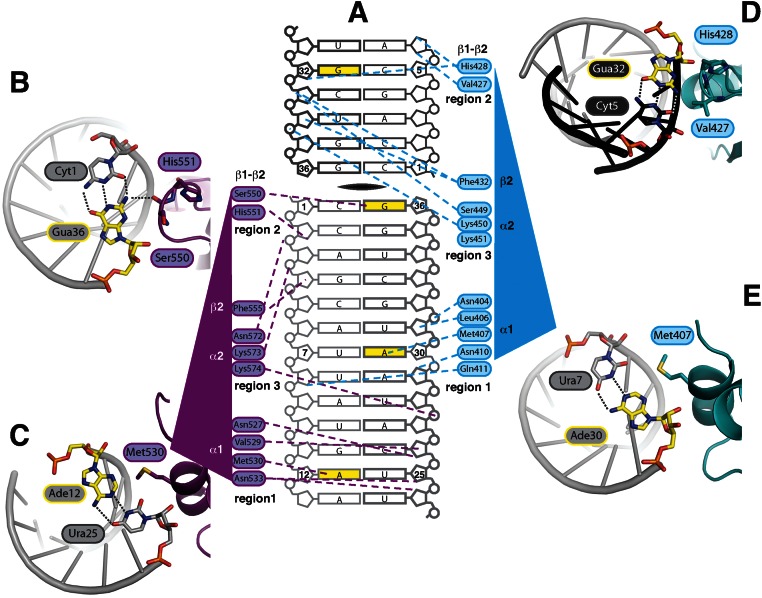
Base-specific interactions of NF90_dsRBDsΔNLS_ in the 18mer dsRNA minor groove. (**A**) Schematic of 18mer RNA interactions with dsRBDs, coloured as in Figure 1. Blue and purple dotted lines indicate contacts between proteins residues and dsRNA. Strands that were labelled with 2′F are shown with thicker lines. (**B**–**E**) Close-up views of individual interactions with G36, A12, G32 and A30, respectively. Base pairs involved in base-specific interactions are shown as sticks and bases in direct contact with protein are yellow. The view is down the axis of the RNA helix. 2′F modified RNA is modelled and shown as native RNA. Hydrogen bonds are indicated with dotted lines.

Each dsRBD has a classical α1–β1–β2–β3–α2 fold spanning two interfaces with the minor groove (15). The domains complement the shape of A-form RNA and contribute polar and basic side chains to recognize the RNA backbone (Figures 3 and 4). Individual dsRBD domains, consisting of residues 403–467 of dsRBD1 (Figure 3B) and 519–590 of dsRBD2 (Figure 3A) were visible in the electron density map. The root mean square deviation (r.m.s.d.) of each domain with the molecular replacement input models are 0.6 Å over 61 Cα atoms and 0.9 Å over 60 Cα atoms for dsRBD1 and dsRBD2 respectively. This indicates that structural rearrangements do not occur on binding RNA. The linker region connecting the two domains (residues 468–518) is not visible, consistent with the prediction that this region is natively disordered. A short β-hairpin bend formed by residues 519–527 extends the classical dsRBD fold at the N-terminus of dsRBD2. This sequence was not present in the molecular replacement search model and is conserved in NF90 and SPNR dsRBD2 domains (Supplementary Figure S3). The hairpin is stabilized by an interaction between Ile520 and Tyr579 on helix α2 in the core structure of dsRBD2 (using the 3-letter amino acid code, Supplementary Figure S2B).

### NF90 shows additional base-specific interactions in the minor groove

DsRBDs are characterized by three regions of interaction with RNA (Figure 4) (15). Helix α1 contacts the minor groove in Region 1. Region 2 (mediated by the loop between β1 and β2) is a second minor groove interaction. Region 3, at the N-terminus of helix α2, contacts the major groove between Regions 1 and 2. Most interactions made by NF90 with dsRNA are via polar and positively charged side chains that interact with ribose and phosphate groups (Figure 4A), consistent with the charge and shape complementarity of binding observed in other dsRBD–dsRNA complexes (14). While dsRNA containing 2′F modifications has been observed to have A-form RNA geometry, high-resolution structures indicate that water hydrogen bonding patterns are disrupted around 2′F atoms in the minor groove compared with native RNA (29). DsRBD–dsRNA complexes typically show interactions between water molecules, 2′OH groups and residues in Region 1. Ribose-side chain interactions at Region 1 of dsRBD1 (Asn404 and Asn410) and dsRBD2 (Asn533, Val529 and Asn527, Figure 4A) that involve 2′F sites are similar to Region 1 sites in other dsRBD structures (22,39–41). This suggests that 2′F atoms have little impact on the association of dsRBDs with dsRNA, consistent with our ITC measurements. Alterations in the positions of water molecules within the protein–RNA interface may play a role in maintaining these interactions. However, at 3.5 Å resolution we observe few ordered water molecules in the structure.

Interestingly, both dsRBDs of NF90 approach similar base-pairs in the two minor groove interfaces spanned by each domain: a C-G base pair is found at the Region 2 interface and an A-U base pair at the Region 1 interface of dsRBD2 (Figure 4B and C). Similarly, dsRBD1 spans across G-C and U-A base pairs at Regions 2 and 1, respectively (Figure 4 D and E). NF90 makes three base-specific interactions in the four minor groove interfaces contacted in the structure (Figure 4). For dsRBD2, these include a hydrogen bond between the main chain carbonyl oxygen of Ser550 and the amino group of G36 (Figure 4B) while a hydrophobic interaction is made between the side chain of Met530 and A12 (Figure 4C). Met407 on dsRBD1 has a similar hydrophobic interaction with A30 (Figure 4E). At the β1–β2 loop of dsRBD1 Val427, the equivalent residue to Ser550, is in close proximity to G32 in the symmetry-related RNA helix (Figure 4D). However the distance between the main chain carbonyl group and the amino moiety is 4 Å, indicating that a hydrogen bond does not form. As valine has a bulkier side chain than serine, steric hindrance likely prevents access to the minor groove.

### Minor groove base interactions are conserved between NF90 and ADAR2

**Figure 5.**
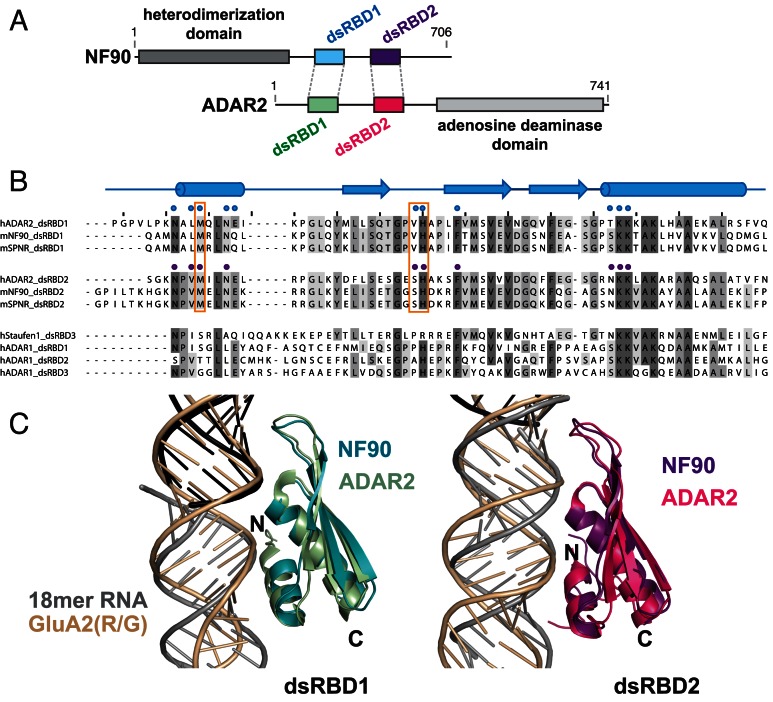
The dsRBDs of NF90 are highly similar to those of ADAR2. (**A**) Schematic of the domain arrangement of NF90 and ADAR2. (**B**) Sequence comparison of dsRBD1 and dsRBD2 domains from human ADAR2, mouse NF90 and mouse SPNR and dsRBDs from human Staufen and ADAR1. Residues involved in RNA binding are marked with blue dots (dsRBD1) and purple dots (dsRBD2). Orange boxes indicate residues involved in specific base recognition interactions. Secondary structure elements are shown above in blue. (**C**) Superposition of dsRBD1 domains (blue and green) and dsRBD2 domains (purple and pink) of NF90 and ADAR2. 18mer RNA is grey while the GluA2(R/G) motif is wheat coloured.

Base-specific readout of the minor groove has previously been observed for the tandem dsRBDs of ADAR2 in a solution structure with GluA2(R/G) editing site hairpin RNA (22). We observed that the tandem dsRBDs of NF90 show high sequence and structural similarity to the tandem dsRBDs of ADAR2 (Figure 5)(22). In the ADAR2–GluA2(R/G) complex, the tandem dsRBDs are bound on one face of the RNA hairpin stem. As in NF90, a linker separates ADAR2 dsRBDs but is a longer, 87 amino acid sequence (Figure 5A and Supplementary Figure S3). Comparison of ADAR2, NF90 and SPNR over the tandem dsRBDs, including the linker, gives an overall sequence identity of ∼50% (Supplementary Figure S3). However, sequence identity >65% is observed when the individual dsRBDs are considered separately (Figure 5B). When dsRBDs are compared between unrelated protein sequences, identity is generally between 20–40% and the least conserved region of the fold is at the N-terminal α1 helix (14) (Figure 5B). Importantly, residues that are involved in base-specific recognition in helix α1 are conserved in ADAR2, NF90 and SPNR but are not conserved in other dsRBDs, including those from ADAR1 and Staufen (Figure 5B).

The similarity between NF90 and ADAR2 is also apparent at the structural level where dsRBD1 from ADAR2 and NF90 superpose with an r.m.s.d of 1.4 Å over 57 Cα atoms and the respective dsRBD2 domains superpose with an r.m.s.d of 1.4 Å over 61 Cα atoms. Interestingly, GluA2(R/G) RNA curves around dsRBD1 of ADAR2 with a gentle deviation from the main helical axis, similar to the kink generated by the intermolecular stacking region of the 18mer dsRNA around its interaction site with NF90 dsRBD1 (Figure 5C).

**Figure 6.**
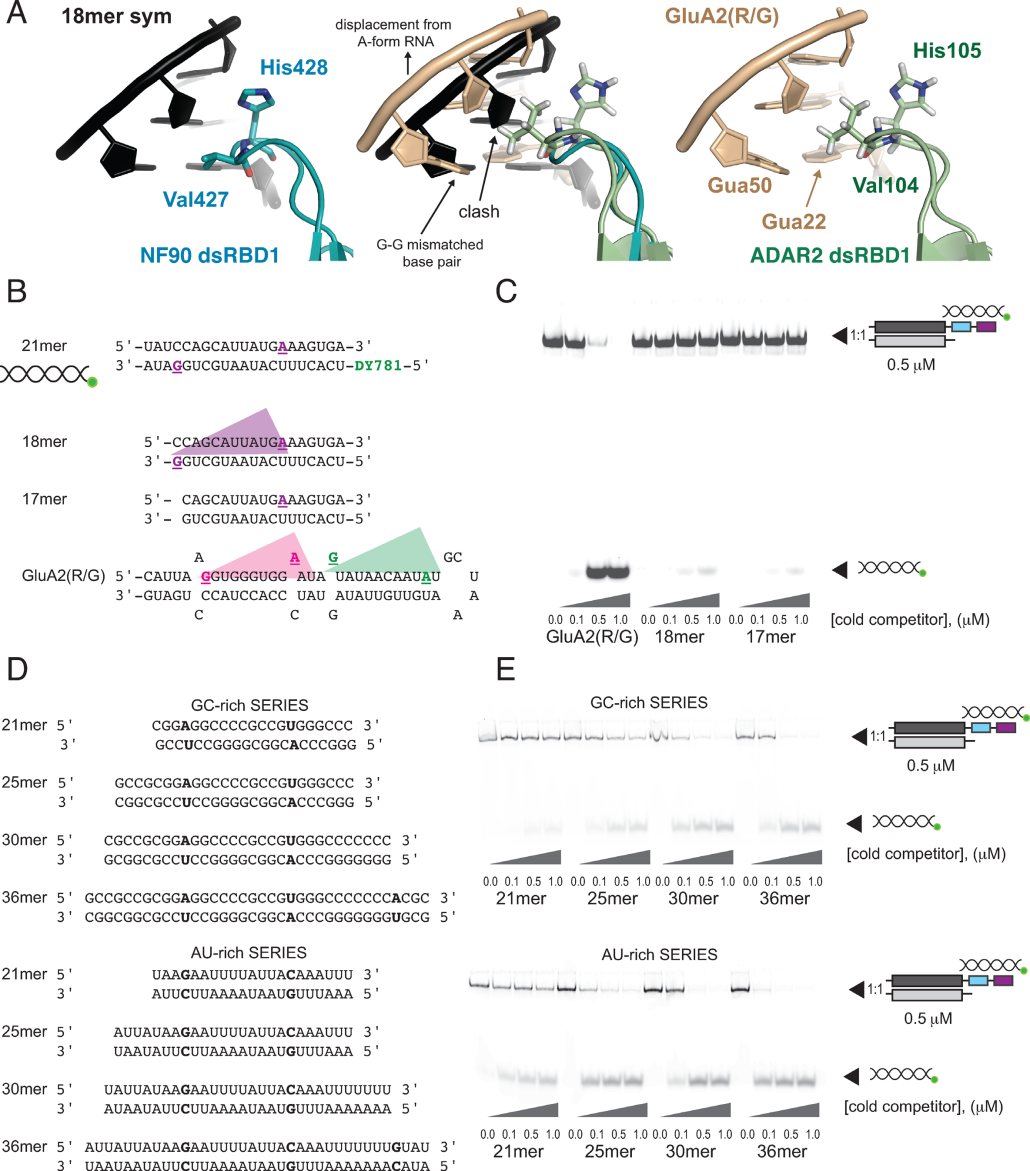
Strict A-form RNA conformation of 18mer prevents the formation of a hydrogen bond with main chain atoms of dsRBD1. (**A**) NF90 Val427 and His428 approach a G-C base pair in 18mer RNA but the side chain of Val427 prevents the formation of a base-specific amino group of the guanine (left). In the ADAR2–GluA2(R/G) complex Val104 makes a closer approach to the minor groove, allowing a specific hydrogen bond to form with guanine 22 (right). Superposition of dsRBD1 from ADAR2 and NF90 (middle panel), shows that the guanine–guanine mismatch in GluA2(R/G) causes a displacement of the RNA backbone from a perfect A-form. In the absence of this displacement Val104 would clash with the ribose group of the subsequent base pair. (**B**) Schematic showing dsRNA species used in competition assay. Specifically recognized bases are bold, underlined and coloured according to the associated dsRBD domain (represented by coloured triangles) based on Figure 4A. NF90 and ADAR2 dsRBDs span across 8, 9 or 10 base pairs. (**C**) Competition assay with a 1:1 complex of NF90_long_/NF45 with DY781-labelled 21mer dsRNA competed by GluA2(R/G) hairpin, 18mer dsRNA and 17mer dsRNA. Concentrations of the unlabelled competitors are indicated below the gel. (**D**) An overview of GC- and AU-rich sequences of increasing length used for competition assays in (**E**). (E) Complexes of NF90_long_/NF45 with labelled 21mer dsRNA were competed with increasing concentrations of GC- or AU-rich dsRNA. Concentrations of the competitors are shown under the gel.

The interactions at the Region 1 and 2 interfaces of NF90 are almost identical to base-specific interactions observed in ADAR2 bound to GluA2(R/G) (22). In this structure, van der Waals contacts between methionine residues and adenine bases are seen in the Region 1 interactions (contributed by Met84_ADAR2_ and Met238_ADAR2_). Hydrogen bonds are formed between main chain carbonyl oxygens in the β1–β2 loop of both ADAR2 dsRBDs (by Val104_ADAR2_ and Ser258_ADAR2_, respectively, Figures 5B and 6A) with amine moieties contributed by guanine bases. The guanine base that hydrogen bonds to the main chain at Val104_ADAR2_ is mismatched with a guanine base on the opposite strand, causing a localized deviation from A-form structure (Figure 6A). In the NF90–RNA complex the dsRNA structure is not disturbed; superposition of ADAR2 dsRBD1 on NF90 dsRBD1 indicates that the deviation from ideal A-form RNA allows Val104_ADAR2_ deeper access into the minor groove, whereas it would clash with the RNA backbone of A-form RNA (Figure 6A). Previous studies have shown that mutating the GluA2(R/G) G-G mismatch to a C-G base pair has little effect on RNA binding by ADAR2 but significantly reduces levels of editing (22,42). Our structure suggests that the subtle, localized deviations from classical A-form RNA frequently observed in ADAR2 substrates may be important for allowing deeper minor groove access for Region 1 of dsRBD1 and thus have an impact on the efficiency of editing.

Given the significant structural similarities between the NF90_dsRBDsΔNLS_–18mer RNA and ADAR2–GluA2(R/G) hairpin complexes, it seems likely that NF90 will recognize ADAR2 substrates. To test this hypothesis we titrated 1:1 complexes of NF90_long_/NF45 pre-incubated with labelled 21mer dsRNA with increasing concentrations of unlabelled GluA2(R/G) as a competitor (Figure 6B and C). As the concentration of GluA2(R/G) increases, 21mer RNA is released efficiently from its complex with NF90_long_/NF45 demonstrating that GluA2(R/G) hairpin does indeed bind to NF90_long_/NF45 complexes (Figure 6C). However, NF90_long_/NF45–21mer dsRNA complexes are not efficiently competed by the 18mer dsRNA used in the crystal structure or with a slightly shorter 17mer dsRNA (Figure 6B and C). All competitors form single species under native conditions (Supplementary Figure S1A).

To explore further the importance of direct base interactions, we carried out competition experiments with either a GC-rich or AU-rich dsRNA of increasing lengths (21–36 bp, Figure 6D). The GC-rich sequence had two or three A-U/U-A base pairs each separated by 10 bases such that there was no position in the duplex where an ideal G-X_10_-A interaction could be made. Similarly, AU-rich sequences had only two or three G-C/C-G base pairs, each separated by 10 bases. In neither case is an ideal G-X_10_-A motif present. If G-X_10_-A motifs are not important for NF90 dsRNA recognition, we would expect both AU-rich and GC-rich 21mers to compete the labelled dsRNA. However, neither 21mer is a good competitor (Figure 6E). We would further expect that if interactions with bases in the minor groove of dsRNA are not important for binding dsRNA, competitors of the same length but different sequence composition would compete equally well with labelled dsRNA. As we increase the length of the cold competitor, labelled dsRNA was competed more efficiently (Figure 6E). This is consistent with dsRBD:dsRNA interactions being dominated by electrostatic and shape complementarity interactions between the protein and the RNA backbone. However, AU-rich sequences showed a greater propensity to displace the labelled dsRNA than GC-rich sequences (Figure 6E). The difference in displacement activity with RNA of different sequence composition supports our hypothesis that NF90 dsRNA binding is influenced by base interactions.

The primary differences between the ADAR2-GluA2(R/G) and NF90 NF90_dsRBDsΔNLS_–18mer RNA complexes are in the spacing of the bases that are recognized and the RNA strand from which they originate (Figure 6B, Supplementary Figure S4). The two sites in GluA2(R/G) that are recognized by ADAR2 have guanine and adenine bases originating from the same RNA strand and separated by 8 or 9 nt. This motif has been denoted as a G-X_n_-A motif where *n* = 8 or 9. In the NF90_dsRBDsΔNLS_–18mer RNA complex, 10 bases separate guanine and adenine bases at both recognition sites, i.e. the motif is G-X_10_-A and the bases emanate from different strands. The difference in spacing arises because 18mer RNA has perfect A-form geometry while the GluA2(R/G) motif is slightly distorted by base mismatches. Differences in the NF90_dsRBDsΔNLS_–18mer RNA complex generated by guanine bases coming from different strands are accommodated by small shifts in conformation of the β1–β2 loop in Region 2 (Figures 3 and 4). Methionine side chains that mediate the base-specific interactions with adenine bases make further accommodations to enable this binding mode. Methionine side chains can access a larger range of rotamers than most side chains and this may contribute to the plasticity of interactions. It has also been noted previously that G-C base pairs are difficult to distinguish from C-G base pairs in the minor groove as the amine group presented by guanine is centrally placed (43). Therefore, it is perhaps not surprising that either orientation of G-X_n_-A can be recognized by the dsRBDs. This shows that the G-X_10_-A motif is has no polarity and implies that a broader range of G-X_n_-A combinations may be accessible to NF90 and/or ADAR2 (Supplementary Figure S4).

SPNR has identical sequences to NF90 within its dsRBD domains (Figure 5), suggesting that SPNR interacts with RNA in the same way as NF90. The linker separating the two SPNR dsRBD domains differs in sequence composition from linkers found in NF90 and ADAR2 and is enriched with serine residues. The lack of conservation in these sequences suggests that their main functional requirement is to be flexible. It is also possible that linkers contain regulatory sites for post-translational modifications or short linear interaction motifs that may differ between these proteins. In human NF90, for example, two phosphorylation sites have been mapped to the linker and a common splice variant in human cells, NF90b, contains a four-residue insertion within the linker (44–46).

## DISCUSSION

The structure of the tandem dsRBDs of NF90 with dsRNA reveals a complex with dsRBDs bound either side of dsRNA. One dsRBD bridges two dsRNA 18mers, inducing a bend where the two dsRNA molecules meet (Figure 2B). We show, using *in vitro* binding assays that NF90 can dimerize on dsRNA although we only observe a 1:1 complex of dsRNA to NF90 in the crystal structure. It is possible that the formation of a 1:2 complex in this system inhibits crystallization. While crystallization of this complex was dependent on the presence of a modified RNA strand, containing 2′F at each position, this modification does not seem to have altered the binding constant of this interaction or hindered the association of the protein with the modified ribose moieties. This suggests that 2′F modifications might be a useful tool to promote crystallization of other protein–RNA complexes.

Both dsRBD1 and dsRBD2 interact across two minor groove surfaces of RNA that have G-C/C-G and A-U/U-A base pairs separated by 10 base pairs. This binding mode is highly similar to that observed in complexes of the RNA editing enzyme ADAR2 with a physiological substrate, the 71 nt GluA2(R/G) hairpin motif. Serendipitously, the structure of NF90_dsRBDsΔNLS_–18mer RNA gives an insight in to ADAR2 sequence requirements for dsRNA recognition. Comparison with our structure shows how subtle deviations from ideal A-form RNA such as RNA curvature; localized disruptions by a G-G mismatch; and overwinding of the RNA helix inherent in the GluA2(R/G) motif facilitate interactions with ADAR2. As with NF90, several reports have suggested that ADAR2 is active as a dimer, although whether dimerization is dsRNA-dependent is currently a matter of debate (47–50).

It is notable that ADAR2 side chains required for base-specific recognition are conserved in vertebrates but are less well conserved in Drosophila (Supplementary Figure S3). NF90 and SPNR are vertebrate-specific proteins. The nearest relative in fruit flies is the zinc finger protein, Zn72D, a homologue of the vertebrate protein Zinc Finger RNA-binding protein, Zfr. Zn72D contains an NF45-heterodimerization domain like that found in NF90 and SPNR but does not encode dsRBD domains (13). It is seems likely that NF90 and SPNR arose in vertebrates from a domain-shuffling event that combined ADAR2 dsRBD domains with the NF45-heterodimerization domains from Zfr.

**Figure 7.**
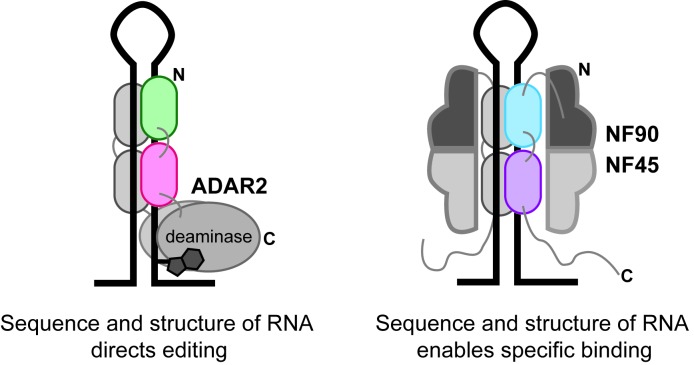
Comparison of dsRNA binding modes by ADAR2 and NF90/NF45. ADAR2 recognizes dsRNA as a homodimer and deaminates structured RNA substrates. NF90/NF45 likely uses similar structural and sequence constraints to recognize specific cellular RNAs. Homodimerization of NF90/NF45 complexes extends the binding surface for RNA recognition.

That NF90 and ADAR2 share a similar mode of binding on dsRNA gives an insight into NF90 function. An important aspect of ADAR2 function is the ability to direct A-to-I editing at a specific residue and the dsRBD domains play a critical part in substrate selection. While we do not yet understand the full physiological implications of this similarity, our data suggest that, like ADAR2, the binding of NF90 on endogenous dsRNA targets may be influenced by sequence elements as well as structural elements (Figure 7). This would be consistent with its proposed role in post-transcriptional control of gene expression. This contrasts with dsRBD-containing proteins involved, for example, in miRNA biogenesis such as Dicer and DGCR8, where an important function of these proteins is to recognize common structural elements of RNA molecules with diverse sequences. It is tempting to speculate that NF90/NF45 might play a regulatory role in RNA editing, perhaps as a direct competitor to ADAR2. To our knowledge there are no reports that NF90 affects ADAR2 editing, although an interaction between NF90 and ADAR1 has previously been reported (51). An analysis of the role of NF90/NF45 and related proteins in regulating RNA editing awaits future functional studies.

## ACCESSION NUMBER

Data have been deposited in the PDB with the following accession code: 5DV7.

## Supplementary Material

SUPPLEMENTARY DATA
