# Supplementary material for: Nuclear factor 90 uses an ADAR2-like binding mode to recognize specific bases in dsRNA
Source: Nucleic Acids Res. 2015 Dec 27;44(4):1924–36. doi: 10.1093/nar/gkv1508 (PMC4770229; doi:10.1093/nar/gkv1508)
Supplement: SUPPLEMENTARY DATA [file supp_44_4_1924__index.html]

Nuclear factor 90 uses an ADAR2-like binding mode to recognize specific bases in dsRNA — Nuclear factor 90 uses an ADAR2-like binding mode to recognize specific bases in dsRNA — SUPPLEMENTARY DATA 

# Nuclear factor 90 uses an ADAR2-like binding mode to recognize specific bases in dsRNA

## SUPPLEMENTARY DATA

- SUPPLEMENTARY DATA
