# Supplementary material for: Nuclear factor 90 uses an ADAR2-like binding mode to recognize specific bases in dsRNA
Source: Nucleic Acids Res. 2015 Dec 27;44(4):1924–36. doi: 10.1093/nar/gkv1508 (PMC4770229; doi:10.1093/nar/gkv1508)
Supplement: SUPPLEMENTARY DATA [file supp_gkv1508_nar-02134-r-2015-File009.pdf]

Supplementary Figures for Jayachandran et al.

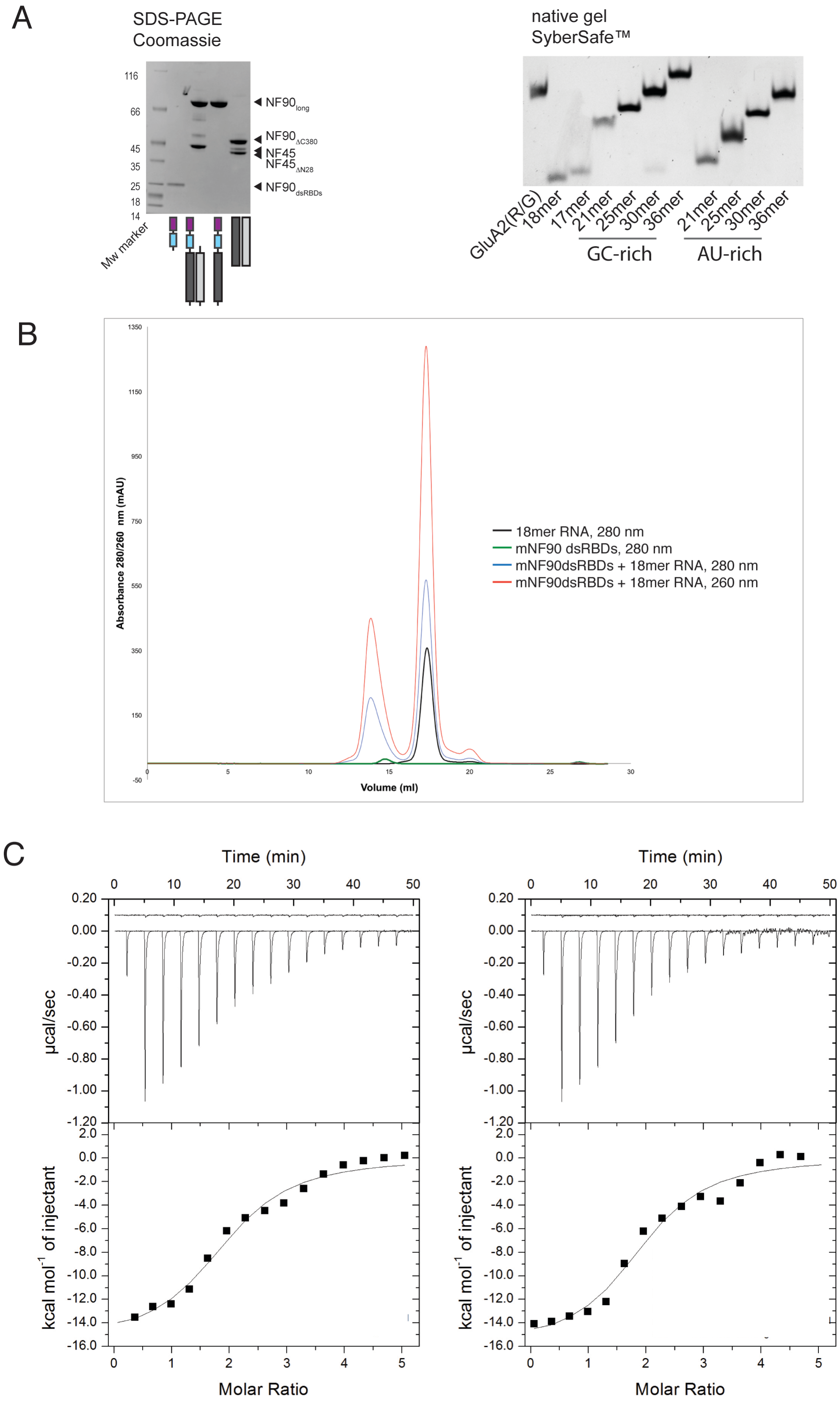

Binding reaction	$K_{d,\,app}$ mean ± s.d.
$K_{D,\,app}$ $NF90_{dsRBDs\Delta NLS}$ +18mer RNA unmodified	2.40 µM ± 0.25 (n=3)
$K_{D,\,app}$ $NF90_{dsRBDs\Delta NLS}$ +18mer RNA 2'F on one strand	2.38 µM ± 0.85 (n=3)

Figure S1 - Formation of the $NF90_{dsRBDs}$ complex with 18mer RNA

A Quality control of protein and RNA components used in RNA binding assays. SDS-PAGE gel of proteins is shown for $NF90_{dsRBDs}$, $NF90_{long}/NF45$, $NF90_{long}$ and $NF90_{\Delta C380}/NF45_{\Delta N28}$. A SyberSafe-stained native gel of dsRNA constructs is also shown.

B   Analytical size exclusion chromatogram showing elution profiles of equal concentrations of 18mer RNA (black trace: absorbance at 260 nm), $mNF90_{dsRBDs}$ (green trace: absorbance at 280 nm) and a 1:1 complex of the two components (blue trace: absorbance at 280 nm, red trace: absorbance at 260 nm). Note that $mNF90_{dsRBDs}$ does not contain any tryptophan residues and so absorbs poorly at 280 nm.

C   Typical ITC reactions with $NF90_{dsRBDs\Delta NLS}$ and 18mer RNA, formed with either native or 2'F modified RNA. A control reaction of protein titrated into buffer is shown at the top.

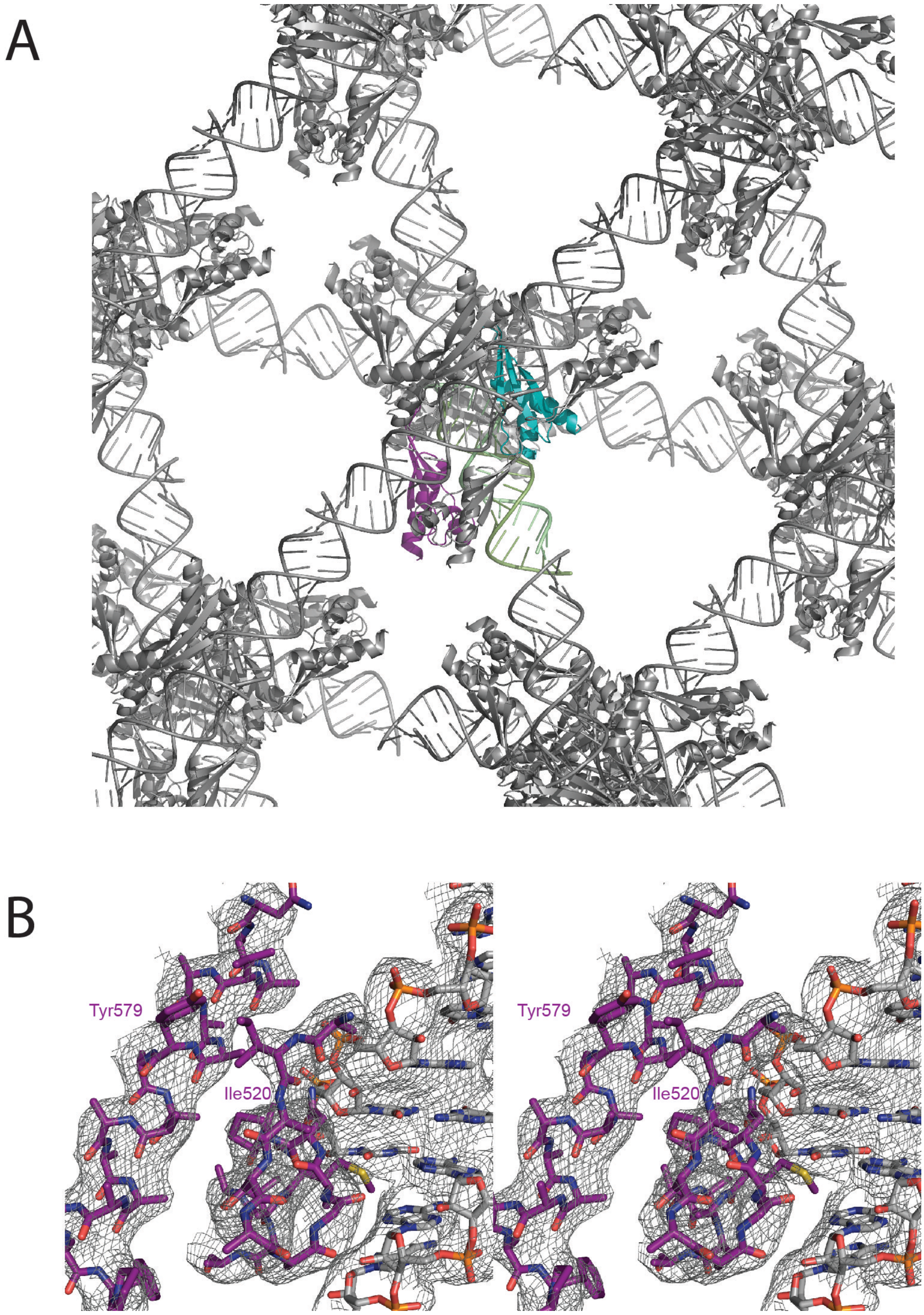

Figure S2 - Symmetry and stereo views of $NF90_{dsRBDs}$ with 18mer RNA in the crystal

A An overview of the symmetry arrangement in crystals of $mNF90_{dsRBDs}$ + 18mer RNA viewed down the 3-fold symmetry axis. The protein domains in the asymmetric unit are colored as in Fig 1, while the 18mer RNA is colored green. Symmetry equivalent structures are shown in gray.

B Stereo view of the interaction between dsRBD2 (purple) and 18mer RNA, showing the N-terminal extension to this domain that was not in the original search model.

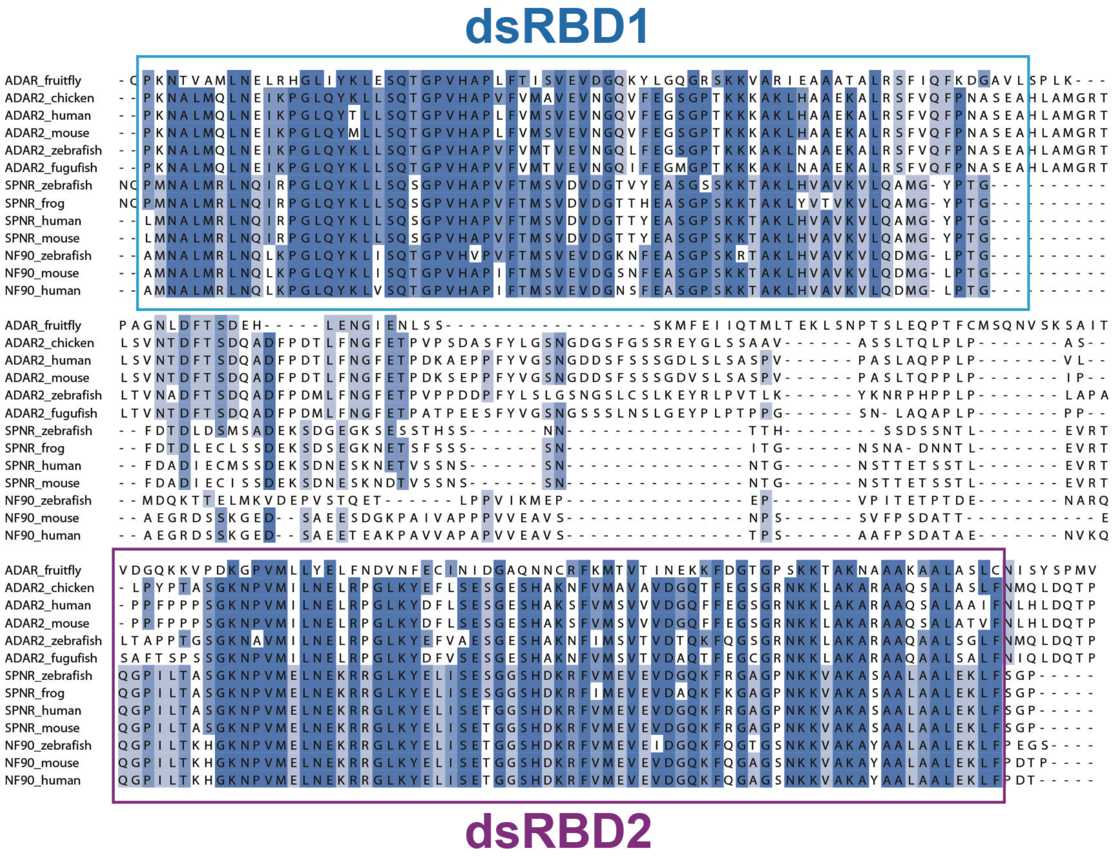

Figure S3 - Multiple sequence alignment of tandem dsRBDs derived from ADAR2, SPNR and NF90 homologues. Note that the linker sequences that separate the dsRBDs are not well conserved either in length or in amino acid composition. In all homologues, this linker sequence is predicted to be natively unstructured.

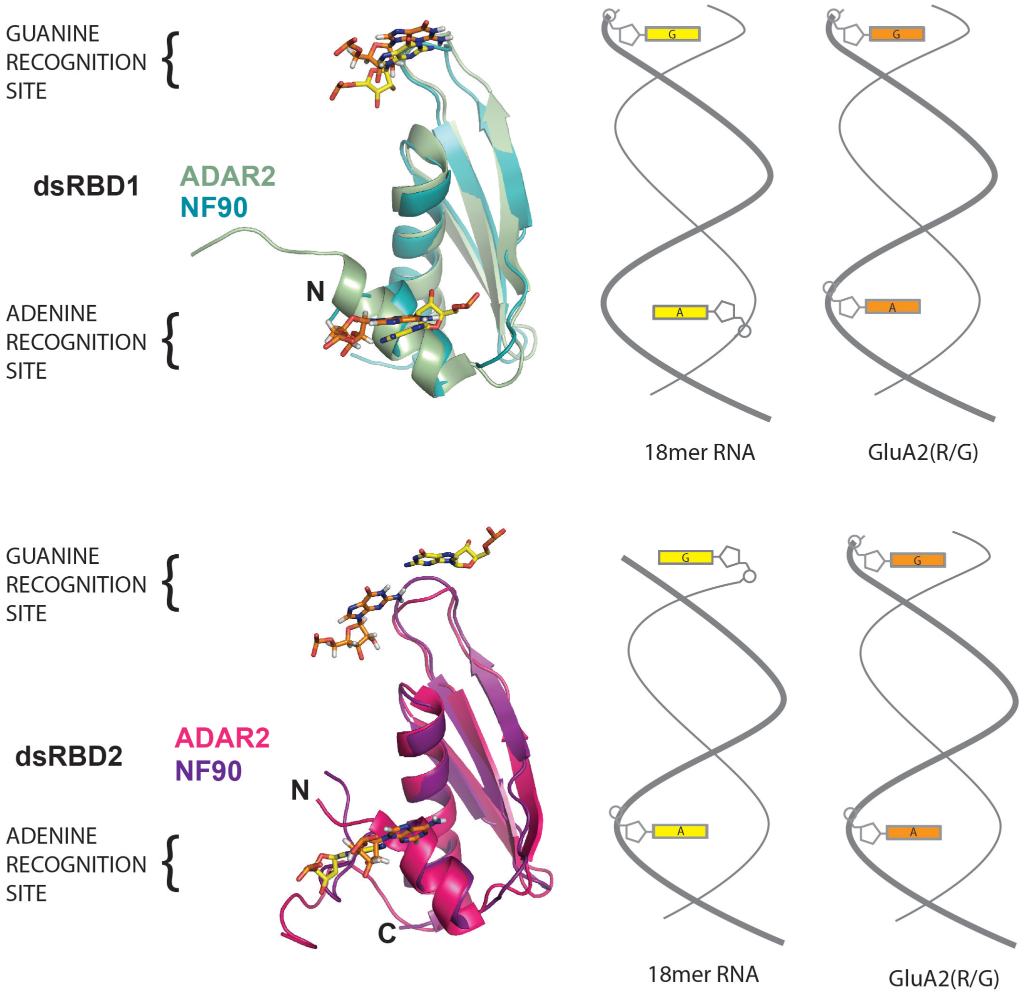

Figure S4 - Superposition of NF90 (cyan, purple) and ADAR2 (green, pink) dsRBD domains viewed from the center of the RNA helix. The RNA has been removed except for stick representations of the bases that are specifically recognized by NF90 (yellow) and ADAR2 (orange). Schematic representations show the origin of the bases that form the G-X_n-A recognition motifs, where G and A are encoded on a continuous stretch of RNA in GluA2(R/G) but not in 18mer RNA.
